# Persistent *Bovine Viral Diarrhea Virus* Infection in Domestic and Wild Small Ruminants and Camelids Including the Mountain Goat (*Oreamnos americanus*)

**DOI:** 10.3389/fmicb.2015.01415

**Published:** 2016-01-07

**Authors:** Danielle D. Nelson, Jennifer L. Duprau, Peregrine L. Wolff, James F. Evermann

**Affiliations:** ^1^Department of Veterinary Microbiology and Pathology, Washington State UniversityPullman, WA, USA; ^2^Nevada Department of WildlifeReno, NV, USA; ^3^Department of Veterinary Clinical Sciences, Washington State UniversityPullman, WA, USA

**Keywords:** *bovine viral diarrhea virus*, mountain goats (*Oreamnos americanus*), small ruminants, persistent infection, wildlife diseases, goats

## Abstract

*Bovine viral diarrhea virus* (BVDV) is a pestivirus best known for causing a variety of disease syndromes in cattle, including gastrointestinal disease, reproductive insufficiency, immunosuppression, mucosal disease, and hemorrhagic syndrome. The virus can be spread by transiently infected individuals and by persistently infected animals that may be asymptomatic while shedding large amounts of virus throughout their lifetime. BVDV has been reported in over 40 domestic and free-ranging species, and persistent infection has been described in eight of those species: white-tailed deer, mule deer, eland, mousedeer, mountain goats, alpacas, sheep, and domestic swine. This paper reviews the various aspects of BVDV transmission, disease syndromes, diagnosis, control, and prevention, as well as examines BVDV infection in domestic and wild small ruminants and camelids including mountain goats (*Oreamnos americanus*).

## Introduction

*Bovine Viral Diarrhea Virus* (BVDV), an RNA virus, is a pestivirus in the family Flaviviridae. Other pestiviruses include *Border Disease Virus* (BDV) in sheep and *Classical Swine Fever Virus* (CSFV) in swine, and more new pestiviruses are being discovered. Though the preponderance of BVDV research has been focused on the primary host, domestic cattle (*Bos taurus)*, there is increasing evidence that the virus infects and causes persistent infection in a wider range of species, including mountain goats (Nelson et al., 2008) and domestic goats (Bachofen et al., 2013). This paper aims to review reports of non-bovine persistently infected (PI) animals, including the mountain goat (*Oreamnos americanus*), and evaluate the implications of wildlife reservoirs of BVDV infection and its impact on the cattle industry.

## Transmission

There are multiple methods of BVDV transmission; the virus can spread horizontally within a herd as well as transmit vertically from cow to calf. Horizontal transmission can occur via transiently infected (TI) animals that shed virus during acute infection. Horizontal transmission can also occur due to PI animals that shed virus throughout their lifespan in all bodily secretions (nasal and ocular discharges, milk/colostrum, semen, urine, and feces; Van Campen and Frolich, 2001). Experiments show that BVDV environmental survival is dependent upon temperature and moisture levels with a maximum survival in bovine farm slurry at 5°C for 3 weeks and at 20°C for 3 days (Botner and Belsham, 2012). There are anecdotal and experimental reports of indirect BVDV transmission from contaminated pens, rectal examination gloves, hypodermic needles, nose tongs, and ambient air (Niskanen and Lindberg, 2003). Experimental vector transmission from PI animals has been successful using horse flies *(Haematopota pluvialis)* and stable flies *(Stomoxys calcitrans)*, but not horn flies *(Haematobia irritans*, Chamorro et al., 2011). Since studies of indirect transmission can be difficult to adequately control, repeated studies with strict controls are necessary to determine and confirm the many possible indirect routes of BVDV transmission.

A PI animal occurs when the fetus is exposed to BVDV in the first or second trimester (45–125 days), prior to maturation of its immune system. In these feti, the virus is recognized as self, resulting in an immune-tolerant state and persistent viremia without seroconversion. However, if a different strain of the virus infects the PI animal (superinfection), they can immunologically respond, resulting in seropositivity (Walz et al., 2010). Vertical transmission may occur from a PI dam *in utero* to her offspring. In vertical transmission, the outcome of infection is determined by the stage of fetal maturation when exposed to the virus *in utero*. If the fetus is infected in the first trimester, it will likely abort, mummify, or show a variety of congenital defects. Infection during the second trimester results in a PI animal, as previously discussed. By the third trimester of gestation (>180 days), the fetus is immune-competent and will mount an immune response that may result in abortion, or the birth of a healthy or weak and seropositive calf. BVDV virus can be transmitted from PI or TI animals through direct contact, shared feed and water sources, environmental contamination, frozen embryos or semen, *in utero*, or fomites (Thurmond, 2005).

Nettleton (1990) stated that “the probability exists, therefore, that pestiviruses have evolved along with their own host species. Interspecies transmission is achieved easily experimentally and it is prudent to believe that it will occur readily in domestic and free-living ruminants when permitted to do so by new husbandry practices or changes in population dynamics.” The pestiviruses are known to cross animal species from both experimental and natural studies (Van Campen and Frolich, 2001). The question that has emerged is how pathogenic are the viruses when they spill over to another animal host (DeFilippis and Villarreal, 2000)? Once interspecies transmission occurs is there intraspecies spread, which propagates the infection in the spillover population?

**Figure 1:**
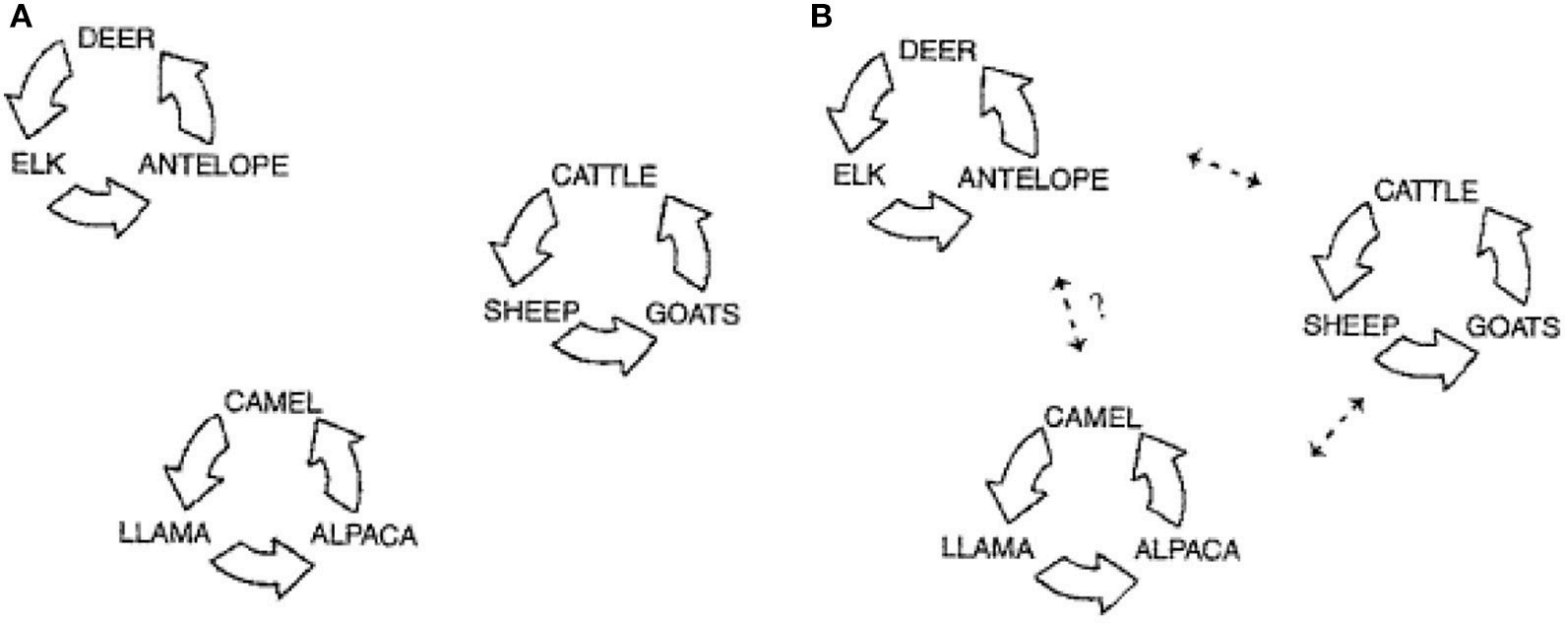
**Schematic depicting the three population groupings for pestivirus infections**. **(A)** Represents the circulation of the virus infection within three distinct main host clusters: wildlife, domestic livestock, and camelids. **(B)** Represents the documented spread of virus between these clusters, and the potential for transmission between the camelid cluster and wildlife cluster (modified from Evermann, 2006).

In the majority of cases involving llamas and alpacas there has been some commingling with cattle, sheep, or goats. The consensus has been that there is spillover of pestiviruses, primarily BVDV, from cattle to llamas and alpacas (Belknap et al., 2000). Levels of BVDV in viremic cattle that are PI are very high, >10^4^ TCID_50_/0.1 mL (Brownlie et al., 2000). This would make them prime candidates for shedding to susceptible llamas and alpacas. However, if the infecting virus did not replicate well, or the immune response was elevated, then further intrahost spread would not likely occur. This latter observation appears the best explanation to date. However, serologic data from camels (Evermann, 2006), and wildlife, including roe deer (Fischer et al., 1998), strongly suggest that unique pestiviruses are infecting these species independent of cattle, sheep and goats. This would indicate that there are several host clusters (Figure 1A) in which strains of pestivirus are circulating within the cluster. Given optimum conditions such as BVDV PI animals, commingling stress, temporarily immunosuppressed pregnant animals, pestivirus naïve animals, and virulent pestivirus strains, then pestivirus transmission may occur between clusters (Figure 1B). Following infection, disease may occur, but rarely would an epidemic develop since intrahost spread would likely be negligible (Mattson, 1994; DeFilippis and Villarreal, 2000).

## Disease syndromes

*Bovine viral diarrhea virus* is known for causing a variety of disease presentations in cattle and other ungulates. There are two genotypes of the virus: BVDV-1 and BVDV-2, both of which have also been isolated from non-bovine species. The genotypes are further divided into cytopathic (CP) and non-cytopathic (NCP) subtypes. CP BVDV arises from rare mutations of the NCP strains. NCP viruses are associated with the majority of BVDV infections (90%) and cause mild transient infection as well as persistent infection. CP biotypes cause severe acute and peracute transient disease as well as mucosal disease in superinfected PI animals (Walz et al., 2010). In general, transient BVDV infections can be divided into five categories: acute, severe acute, hemorrhagic infection, bovine respiratory disease, and immunosuppression-only. In addition to these five syndromes, BVDV can also cause chronic disease and mucosal disease in PI animals (Evermann and Barrington, 2005). PI animals may be subclinically infected or may be runted with ill thrift. The importance of acute (transient) infections in the transmission and maintenance of BVDV within a population of animals (domestic and wild) should not be underestimated. These TI animals are responsible for up to 93% of all *in utero* infections that result in the birth of PI calves (Wittum et al., 2001).

## What is the role of BVDV persistent infection in wild ungulates?

Although BVDV is named for its primary host, its prevalence in non-bovine species has become increasingly recognized. To date, the virus has been isolated in over 40 species and serological evidence indicates that most wild ruminants are susceptible to BVDV infection. In addition to wildlife, multiple domestic non-bovid species have also been reported to carry and spread the disease, including sheep, goats, new world camelids, and swine. There is evidence of transient infection within most of these species, resulting in the familiar BVDV syndromes of reproductive insufficiency, respiratory disease, and immunosuppression (Carman et al., 2005; Vilcek and Nettleton, 2006; Ames, 2008; Nelson et al., 2008). However, a select few have been proven to become PI with the virus and act as a significant transmission source to other susceptible species. Natural or experimental persistent infection has been reported in mountain goats (Nelson et al., 2008) and domestic goats (Bachofen et al., 2013), domestic sheep (Scherer et al., 2001), swine (Terpstra and Wensvoort, 1997), alpaca (Mattson et al., 2006), eland, mule deer, white-tailed deer, and mousedeer (Duncan et al., 2008). Since PI animals represent the greatest risk for disease transmission, the identification of PI wildlife species is cause for concern.

In most cases of infection with BVDV in non-bovid species, the primary source of virus is unknown, though the virus exposure is assumed to stem from initial spillover from cattle. This spillover can occur through multiple routes, including direct contact, aerosol, environmental contamination, or fomite transmission, such as shared feed and water sources or shared equipment (Ames, 2008). Direct contact and shared environment are important sources for cattle producers to consider when attempting to eliminate BVDV-associated disease from their herds, while shared equipment is a more important consideration in captive wildlife collections.

In a USDA agricultural census, over 60% of dairy and over 70% of beef producers reported direct contact of their stock to wild cervids (USDA National Agriculture Statistics Service, 2007). Of the potential PI wildlife species, white-tailed deer *(Odocoileus virginianus)* likely present the greatest threat to livestock producers due to their wide range and adaptability to dairy or ranching management systems. Deer are commonly in close contact to cattle, often sharing feed, water, and lounging sites. This environmental interaction between species serves as a source of BVDV transmission. Multiple studies have examined the interactions between white-tailed deer and cattle and how the virus transmits between the two species (Passler et al., 2007; Duncan et al., 2008; Passler and Walz, 2009). One experiment housed seven pregnant white-tailed deer with two known PI cattle to test whether the virus would transmit through cohabitation. Though the does and cows were not observed in physical contact, feed and water sources as well as lounging areas were shared between species. Of the nine live fawns born, all three singlet fawns were born PI (virus positive, antibody negative), and all twin fawns cleared the infection and were born virus negative and seropositive (Passler and Walz, 2009).

Another North American cervid known to have the potential for persistent infection is mule deer *(Odocoileus hemionus)*. Thus, in a routine survey of tissues from hunter-harvested deer in Colorado for chronic wasting disease, BVDV was added to the testing protocol. A single adult male mule deer was positive on skin immunohistochemistry (IHC), which is a consistent finding in BVDV PI cattle. The animal showed no signs of illness, though the virus was identified in both the submitted ear and lymph node, and these findings are suggestive of persistent infection. PCR was performed to further characterize the virus as BVDV-1. The source of infection in this case was unknown, but is assumed to be spillover from cattle (Duncan et al., 2008).

In 2000, a survey was performed on 1539 eland *(Taurotragus oryx)* in Zimbabwe to assess the number of animals infected with BVDV in a high density cattle area, and 32% of eland sampled were antibody positive on ELISA. Three animals were found to have NCP BVDV. Two of these seroconverted on subsequent sampling dates, but one young female remained viremic over time and was determined to be persistently affected. The PI animal eventually lost condition and died following an episode of febrile illness (Vilcek et al., 2000). Presumably domestic cattle served as the primary viral source.

There are multiple reports of PI domestic species including new world camelids. Two PI crias were identified on a Pennsylvania breeding farm. Both presented for stunting and immunosuppression. The first affected cria was determined to be PI following repeated virus isolation in the absence of seroconversion. Euthanasia was elected to protect the breeding herd. The second cria was euthanized after initial virus isolation at 6 weeks of age. On the same day, blood was submitted from the 15 adults on the property. 14/15 were seropositive and one additional male was transiently viremic (Mattson et al., 2006).

A second report in alpacas chronicled a farm in eastern Ontario, Canada, where a chronically ill cria was evaluated at necropsy. Upon reviewing the herd records, it was discovered that the cria's dam had traveled to four different breeding farms during her pregnancy, two of which had experienced numerous abortions and stillbirths. The Ontario herd experienced vague herd illness following the birth of the PI cria, characterized by anorexia, lethargy, and several abortions (all of which were positive for BVDV). Out of 20 animals tested, 17 were positive for antibody to both BVDV strains. Each of the 13 crias born after the initial abortion were tested at birth for BVDV using RT-PCR. Only one of these crias was positive, euthanasia was elected. The euthanized cria was positive for virus at necropsy using IHC (Carman et al., 2005).

*Bovine viral diarrhea virus* infection has also been reported in domestic sheep. Ewes were experimentally infected with NCP BVDV-2 at three different stages of gestation. Of 19 ewes infected at 50–60 days of gestation, there was a 77% fetal death loss. The lambs that were born alive were positive for BVDV at birth and were negative for antibody after maternal antibody waned, which confirms PI status. Of the ewes infected at 65–70 days gestation, the death loss was 66.6%, and the live lambs were virus negative and antibody positive, demonstrating an appropriate immune response to viremia *in utero*. Ewes infected with BVDV at 120–125 days of gestation gave birth to healthy virus negative, antibody positive lambs (Scherer et al., 2001).

Domestic swine can also become PI. Terpstra and Wensvoort published a case in which a litter of piglets became infected with BVDV. Of the 13 pigs in the litter, seven died within 2–4 weeks of birth. Three of the remaining six were euthanized due to wasting and deep ulcers of the jaw and extremities. Three of the remaining pigs survived until slaughter, including one PI boar, one seropositive boar, and one intersex pig. The PI boar remained viremic and immunotolerant until slaughter at 26 weeks of age. The viremic boar shed the virus in oropharyngeal fluid, urine, and semen and was leukopenic from 3 months onward (Terpstra and Wensvoort, 1997). Since persistent infection can occur in domestic swine, feral swine can potentially become sources for disease transmission.

In 2005, routine quarantine at the Copenhagen zoo revealed a BVDV PI Lesser Malayan mousedeer *(Tragulus kanchil)*, prompting a trace back to be performed on the deer's lineage. The trace back identified 10 PI animals in two generations all of which could be traced back to a single PI female. All other animals in contact with these PI mousedeer were found to be antibody positive and virus negative. The PI mousedeer was asymptomatic throughout the testing, but was viremic over multiple testing dates without evidence of seroconversion (Uttenthal et al., 2006). This is the first report of the existence of mature PI animals, other than cattle, that were able to reproduce and produce PI offspring.

Domestic goats can be infected with BVDV with reproductive disease as the most common disease manifestation. PI cattle are considered the main source of infection, as this occurs under natural and experimental conditions. Pregnant goats in direct contact with a PI calf aborted or produced PI kids, and pregnant goats in contact with PI kids produced additional PI kids (Bachofen et al., 2013). Another similar study with pregnant goats exposed to PI heifers with BVDV-2a resulted in abortion and stillbirth but not PI kids suggesting that the development of PI kids is relatively rare (Broaddus et al., 2007). Another study, produced similar results with intranasal inoculation of pregnant goats with BVDV-1 or BVDV-2 resulting in reproductive loss and, less commonly, PI kids further suggesting that BVDV may be maintained in goat populations (Passler et al., 2014). While a native Korean goat developed diarrhea due to BVDV-2 infection (Kim et al., 2006), reports of goats with non-reproductive disease associated with BVDV infection are rare.

There is also evidence for BVDV infection in wild goats. Antibodies to BVDV have been detected in serosurveys of wild mountain goats in Canada (Garde et al., 2005), and wild Alpine and Iberian ibex in Europe (Fernandez-Sirera et al., 2011). There is also direct evidence for BVDV-1 and BVDV-2 infection in mountain goats. Mountain goats in Nevada experienced an all age bacterial bronchopneumonia die off during the winter of 2009–2010 and three sampled mountain goats from this outbreak were seropositive for BVDV-1 and BVDV-2 on virus neutralization. In 2011, one mountain goat kid from the same area also died of bronchopneumonia with suppurative mural enteritis and suppurative serositis suggesting secondary septicemia. Necrotizing mesenteric lymphadenitis prompted testing for BVDV infection. Though BVDV IHC was negative, virus isolation of spleen was positive for BVDV1a confirming current natural infection in a free-living mountain goat (Wolff et al., in press).

To the authors' knowledge, mountain goats are the only wild goat species with definitive evidence of persistent pestivirus infection. Two captive mountain goats from a zoological collection in Idaho were proven to be infected with BVDV-2. While one goat had evidence of systemic BVDV-2 infection by IHC, virus isolation, and PCR with sequencing, the histological lesions indicated that suppurative enteritis with bacterial septicemia was a major factor in the cause of death. Longitudinal evidence of prolonged BVDV infection was not possible in this goat, but persistent infection was considered probable due to prolonged seronegativity and widespread virus distribution without associated necrosis. The second goat from the same premises had suppurative bronchopneumonia and suppurative hepatitis indicating bacterial septicemia was again the likely cause of death. This second goat had repeated longitudinal evidence of BVDV-2 infection by virus isolation and PCR with sequencing yet was seronegative over time providing definitive proof of persistent infection (Nelson et al., 2008).

The epidemiology and spectrum of disease syndromes due to bovine viral diarrhea infection in mountain goats is currently not well understood. Serosurvey of the Idaho zoological collection cohorts (including domestic sheep, domestic goats, mule deer, and whitetail deer in the same pen) suggested there may have been transmission between these wild caught mountain goats and the other ruminants, but the origin of this virus was not determined (Nelson et al., 2008). Evidence of BVDV infection in domestic cattle and free-living bighorn sheep, mountain goats, and mule deer sharing the same range in Nevada demonstrated interspecies transmission in wild settings (Wolff et al., in press). Since pestivirus infection causes immunosuppression with increased susceptibility to bacterial infection, BVDV likely played this indirect role in these mountain goats with septicemia. BVDV infection in mountain goats likely affects reproductive rates as seen in domestic goats and may cause diarrhea as seen in Korean goats (Kim et al., 2006), but this has not been proven. The difficulty of access and limited numbers of these high mountain dwellers will limit further investigations into the incidence, epidemiology, and full characterization of natural disease.

## Control and prevention

Control and prevention of BVDV is based on three elements: elimination of PI animals, biosecurity, and early detection. Many Scandinavian countries are considered BVDV-free following widespread eradication programs in the 1990s based on these elements (Stahl and Alenius, 2012). The methods used to eradicate the disease included identification of positive herds, implementation of quarantine protocols, elimination through rigorous test and cull strategies, and prevention of BVDV introduction into non-infected herds.

Considering that PI animals provide a significant source for virus transmission, the key factor in BVDV control is identification and elimination of PIs. The Swedish eradication program identified PIs by performing serology on virus positive herds to find seronegative animals. Once seronegative animals were detected, virus isolation was performed. If an animal was found to be seronegative and virus positive, it was declared a PI and was eliminated from the herd (Stahl and Alenius, 2012).

Once BVDV is eliminated from the herd, rigorous biosecurity programs should be established to prevent re-introduction of the virus. All incoming animals, including purchased calves, replacement heifers, cows, and bulls, should be tested using one of the methods described above. Three week quarantine practices should also be implemented before introducing newly acquired animals into a disease free herd (Walz et al., 2010). Semen and embryos being used in breeding programs should also be considered as a source for herd infection and only be acquired from BVDV-free sources.

Vaccination may have a role in preventing disease, but efficacy in field conditions is not well documented and practicality is likely limited. Vaccination in domestic livestock is recommended in cases where the risk of re-introduction is high, but should always be used in conjunction with other control methods (Stahl and Alenius, 2012). When a vaccination program is considered, it is important to remember that antibodies associated with vaccination may complicate diagnosis of PI animals. The goal of vaccination is to limit transmission and severity of clinical disease in affected animals, rather than true prevention of BVDV infection. Vaccine use is commonly targeted to prevent the development of PI offspring (Walz et al., 2010).

## Implications for domestic livestock producers

In a 2013 study, seroprevalence of BVDV in cattle was compared to that of white-tailed deer in the state of New York (Kirchgessner et al., 2013). Seroprevalence in cattle herds was found to closely mirror seroprevalence in hunter-harvested white-tailed deer. Given these findings, it is likely that there is an element of spillover/spillback that maintains the disease between these two species. This may also be true of the other species discussed in this paper. In many of the reports (white tail deer, alpaca), the PI animals had known exposure to BVDV positive herds during their gestation. According to a recent USDA survey, over 60% of dairy producers and 70% of beef ranchers report direct contact between their stock and wild cervids (USDA National Agriculture Statistics Service, 2007). Reduced contact between cattle and wildlife can be achieved with non-lethal methods such as high or electrified fencing, livestock protection dogs, enclosing stored feedstuffs, reduction of wasted feed, and elimination of baiting and winter feeding practices (Van Campen and Rhyan, 2009; VerCauteren et al., 2012).

Producers with multiple domestic species on the same premises should be aware of the potential for disease spread among their animals. As discussed earlier, BVDV PIs have been found in sheep, alpacas, and swine. Even if the species are not in direct contact, these PI animals increase the potential for BVDV spread through environmental contamination or use of shared equipment. Biosecurity measures should be implemented for control of disease on mixed-species farms, including decontamination of shared equipment, separation of shared feed or water sources, and reduction of disease spread by personnel tending to multiple species (change clothes, footbaths, hand washing between species). Care should be taken to isolate animals returning from mixed-species exhibitions (fairs, rodeos, shows) upon their return to the breeding herd.

## Implications for wildlife management

As in cattle herds, factors such as population density, adequate habitat/forage, and herd behavior contribute to the number of seropositive susceptible wildlife species in a given area. Though it is an uncommon disease of wildlife, BVDV should be viewed as a threat to the health of wildlife populations and measures should be considered to reduce transmission of the disease within the ecosystem.

For mountain goats, remote high mountain habitat has likely historically minimized BVDV transmission, though as this habitat is increasingly encroached upon by domestic livestock grazing and human development, opportunities for disease transmission are likely increasing over time. Since multiple wildlife species have been shown to be capable of persistent and transient infection, interactions with other wild species such as deer and bighorn sheep also increase the opportunity for disease transmission. Assuming mountain goats are affected similar to domestic goats, BVDV infection likely causes significant reductions in the reproductive rate which could pose additional challenges for this wild species.

Increased surveillance is an important factor in the control and understanding of BVDV infection in wildlife. Implementation of BVDV testing on hunter-harvested samples may be a good way to track disease progression within an ecosystem. The antigen capture ELISA (ACE) test, once validated, could be performed on blood or ear notch samples collected at carcass inspection. It would be a sensitive, specific, and inexpensive way to monitor disease prevalence. PCR could also be performed on pooled samples, as is commonly preferred for diagnosis in cattle herds. Vaccination may eventually become an important consideration for control of BVDV in areas with high disease prevalence in wildlife species.

## Implications for zoological collections

The implications of BVDV in zoological collections are similar to those in the domestic livestock industry. Increased biosecurity practices should be implemented to prevent fomite transmission of the disease between species. Quarantine and testing of new arrivals is also an important consideration for disease control captive collections. Disease transmission should be a consideration when mixed species exhibits are being planned and the animals should be tested accordingly.

## Concluding remarks

*Bovine viral diarrhea virus* is a disease with significant economic and health implications for positive herds. It causes economic losses to producers through loss of production, increased susceptibility to infection, and reproductive insufficiency. Considerable effort has gone into control and eradication of the disease through identification and elimination of PI cattle, but because wildlife and non-bovid have the potential for persistent infection, they must also be considered as an integral part of any eradication effort. Additionally, increased monitoring is an essential part of disease control and identification of new host species. Surveillance for BVDV in wild animal populations is increasing in areas with high seroprevalence and will likely continue to improve. Cattle producers, wildlife conservationists, and zoological staff members all need to consider the role of nonstandard BVDV hosts when attempting to control or eliminate the disease within a population.

## Author contributions

DN is the first author and had the greatest contribution to the research, editing, and writing of this paper. JD is the second author and significantly contributed to the research and writing of this paper. PW is the third author and significantly contributed to the research and writing of this paper. JE is the final author and mentored and editing this paper with some primary writing contribution.

### Conflict of interest statement

The authors declare that the research was conducted in the absence of any commercial or financial relationships that could be construed as a potential conflict of interest.
